# Development of Polyvinyl Alcohol/Kaolin Sponges Stimulated by Marjoram as Hemostatic, Antibacterial, and Antioxidant Dressings for Wound Healing Promotion

**DOI:** 10.3390/ijms222313050

**Published:** 2021-12-02

**Authors:** Tamer M. Tamer, Mosa H. Alsehli, Ahmed M. Omer, Tarek H. Afifi, Maysa M. Sabet, Mohamed S. Mohy-Eldin, Mohamed A. Hassan

**Affiliations:** 1 Polymer Materials Research Department, Advanced Technology and New Materials Research Institute (ATNMRI), City of Scientific Research and Technological Applications (SRTA-City), New Borg El-Arab City, Alexandria 21934, Egypt; Ahmedomer_81@yahoo.com (A.M.O.); Maysamohamed19@yahoo.com (M.M.S.); mohyeldinmohamed@gmail.com (M.S.M.-E.); 2 Department of Chemistry, Taibah University, Madinah 30002, Saudi Arabia; mosa_alsehli@hotmail.com (M.H.A.); afifith@yahoo.com (T.H.A.); 3 Protein Research Department, Genetic Engineering and Biotechnology Research Institute (GEBRI), City of Scientific Research and Technological Applications (SRTA-City), New Borg El-Arab City, Alexandria 21934, Egypt; 4 University Medical Center Göttingen, Georg-August-University, 37073 Göttingen, Germany

**Keywords:** PVA, marjoram oil, kaolin, hemostatic dressing, antibacterial and antioxidant wound dressing

## Abstract

The predominant impediments to cutaneous wound regeneration are hemorrhage and bacterial infections that lead to extensive inflammation with lethal impact. We thus developed a series of composite sponges based on polyvinyl alcohol (PVA) inspired by marjoram essential oil and kaolin (PVA/marjoram/kaolin), adopting a freeze–thaw method to treat irregular wounds by thwarting lethal bleeding and microbial infections. Microstructure analyses manifested three-dimensional interconnected porous structures for PVA/marjoram/kaolin. Additionally, upon increasing marjoram and kaolin concentrations, the pore diameters of the sponges significantly increased, recording a maximum of 34 ± 5.8 µm for PVA-M0.5-K0.1. Moreover, the porosity and degradation properties of PVA/marjoram/kaolin sponges were markedly enhanced compared with the PVA sponge with high swelling capacity. Furthermore, the PVA/marjoram/kaolin sponges exerted exceptional antibacterial performance against *Escherichia coli* and *Bacillus cereus*, along with remarkable antioxidant properties. Moreover, PVA/marjoram/kaolin sponges demonstrated significant thrombogenicity, developing high thrombus mass and hemocompatibility, in addition to their remarkable safety toward fibroblast cells. Notably, this is the first study to our knowledge investigating the effectiveness of marjoram in a polymeric carrier for prospective functioning as a wound dressing. Collectively, the findings suggest the prospective usage of the PVA-M0.5-K0.1 sponge in wound healing for hemorrhage and bacterial infection control.

## 1. Introduction

Wound healing is an inherent rejuvenating reaction to skin damage. Essentially, the cutaneous healing process is orchestrated into four overlapping phases: hemostasis, inflammation, proliferation, and tissue remodeling [1,2].

Instantly following a skin injury, avascular constriction and blood clotting cascade preclude the hemorrhage and infiltration of microorganisms. Furthermore, blood clots also serve as a reservoir for growth factors and cytokines as well as a scaffold for immigrant cells recruited for wound recovery and tissue regeneration [3,4]. On the other hand, bleeding has been the predominant cause of mortality in civilian and military communities over past decades. In trauma patients, instant hemorrhage management through blood clotting intervention is imperative since most deaths emerge within the first hour following severe trauma [5,6]. Moreover, uncontrolled bleeding impedes wound healing and provokes significant consequences, including severe inflammation and microbial infections of the wound [7].

The blood clotting pathway is coordinated in three sequential phases: (I) initiation, which involves the production of thrombin, (II) amplification, which is characterized by activation and aggregation of platelets; and (II) proliferation, which is characterized by the development of fibrin alongside stability of platelet clot. Most applied hemostatic agents play a crucial role by enhancing platelet aggregation and coagulation within the amplification and proliferation phases [8].

A hemostatic agent should encounter specific vital characteristics, including ease of use, cost-effectiveness, hemocompatibility, cytocompatibility, and intrinsic biodegradability [9]. Injectable hydrogels have revealed good healing with hemostatic properties [10]; nevertheless, they typically have insufficient mechanical features, curtailing their implementation as hemostatic materials [11]. Furthermore, such injectable hydrogels might incite adverse complications for patients, aggravating the treated wound since it is painful either to change or remove them, particularly from profound injuries [12]. Thus, developing porous sponges with hemostatic, antibacterial, and antioxidant performances is advantageous because of their distinct three-dimensional structures, which enable them to have mechanical stability. Moreover, it is worth pointing out that the three-dimensional structure fortifies the adhesion and proliferation of cells recruited for wound healing [9].

Polyvinyl alcohol (PVA)-based porous sponges have demonstrated outstanding mechanical properties and remarkable biocompatibility [13]. Physical cross-linking could be adopted to formulate PVA sponges to evade the utilization of chemical cross-linking solvents, which might be hazardous for biomedical applications. Therefore, sequential freeze–thawing could cross-link PVA-based sponges, which could develop crystalline clusters as a cross-linking point [14,15]. Given that the PVA sponge lacks hemostatic and antibacterial capacities, it is thus indispensable to adapt the PVA sponge by incorporating a hemostatic agent, such as kaolin to stimulate blood cells and platelet accumulation, and another bioactive compound (marjoram essential oil) to endow the sponge with antibacterial and antioxidant capacities to frustrate the inflammation in the wound bed.

Kaolin has thus been identified as one of the significant hemostatic agents, which could substantially promote blood coagulation [16]. Kaolin, often known as China clay, principally comprises kaolinite and aluminium silicate [16]. Given that negative charges on the kaolin surface could substantially promote blood coagulation, kaolin was successfully implemented as active material in surgical hemostasis. Furthermore, kaolin could induce factor XII and platelets, further initiating the blood clotting cascade [17].

Marjoram (*Origanum majorana* L.) is one of the promising essential oil candidates for several applications. It is an herbaceous and perennial plant broadly cultivated in various areas of the globe and could be used for traditional medicine [18]. In addition, previous studies have evidenced marjoram’s antibacterial, antioxidant, anti-inflammatory, and anticancer properties [19]. The major constituents of marjoram essential oil are terpinen-4-ol (which accounts for more than 20% of the total), (+)-cis-sabinene hydrate (3–18%), γ-terpinene and terpinolene, thymol, and carvacrol [20]. In addition, the high antioxidant activity of oil derived from marjoram accounted for the predominance of phenolic acids and terpenoids [21].

We presumed that the synergistic effect of the sponge composite based on PVA, marjoram essential oil, and kaolin could effectively frustrate the hemorrhage and the microbial infections of injury alongside the competency of PVA to seal the wound, which could significantly accelerate the action of the devised sponge. We thus fabricated a series of PVA/marjoram/kaolin porous sponge composites as hemostatic and antibacterial wound dressings. The designed sponges were characterized to determine their physical and chemical attributes in order to explore the extent to which these sponges correspond with the paramount traits of the ideal wound dressing. Additionally, the antibacterial and antioxidant activities and the hemostatic capability of the developed sponges were assessed. Moreover, the cytotoxicity of the sponges was studied in relation to the human dermal fibroblast. The findings of these in vitro investigations will ascertain the favorable sponge, which will require further in vitro and in vivo investigations to fully validate its application as a promising hemostatic and antibacterial dressing.

## 2. Results and Discussion

### 2.1. Development of PVA/Marjoram/Kaolin Sponges

In our previous investigations, we manifested the potency of hemostatic and antibacterial performances of PVA/kaolin, which were reinforced by penicillin–streptomycin [22]. However, the antibacterial activities of these sponges substantially depend on the presence of antibiotics that might be frustrated by the emergence of resistant bacteria. Furthermore, the antioxidant activity of these sponges has not been reported.

**Figure 1:**
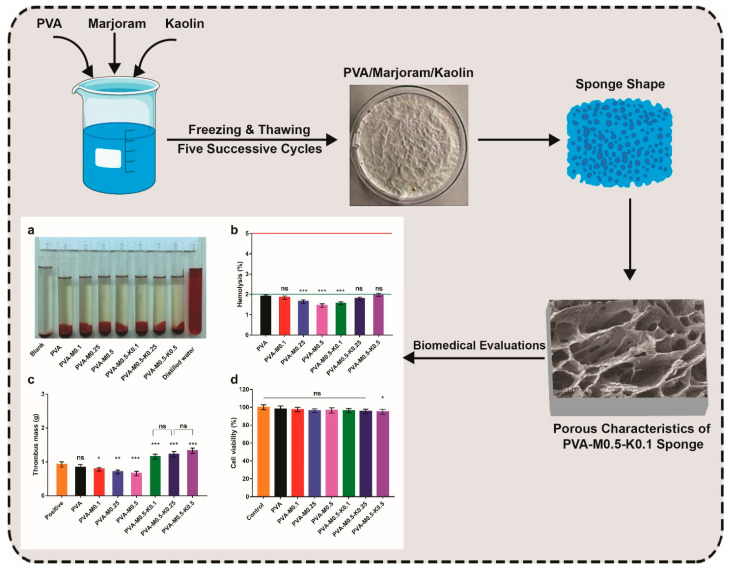
Schematic illustration of the fabrication and biomedical evaluations of hemostatic and antibacterial PVA/marjoram/kaolin sponges. ((**a**–**d**) in the scheme depict some of the biomedical evaluations performed for PVA/marjoram/kaolin composite sponges, including biocompatibility and thrombogenicity with statistical analysis (*** *p* < 0.001, ** *p* < 0.01, * *p* < 0.05, and (ns) points to a non-significant difference).

Due to these shortcomings, we aspired in this study to devise non-antibiotic sponges for further implementation in the wound healing process. Given the particular structure and tunable traits of PVA, it is predominantly recruited for hydrogel development by cyclical freezing–thawing to develop crystalline clusters alongside its competency acting as a carrier of drugs [23]. As portrayed in Figure 1, we formulated new crosslinked sponges using PVA inspired by marjoram extract and kaolin employing a freezing-thawing approach. Moreover, due to the antibacterial, antioxidant, and hemostatic deficiencies of PVA, marjoram extract and kaolin were incorporated into the designed sponges. After freezing-thawing, PVA, marjoram extract, and kaolin, in addition to high water molecules, were entrapped and entangled into the physically crosslinked three-dimensional network of sponges. The developed sponges with various concentrations of marjoram extract were labelled as PVA-M0.1, PVA-M0.25, and PVA-M0.5, while those boosted by marjoram extract and kaolin were denominated as PVA-M0.5-K0.1, PVA-M0.5-K0.25, and PVA-M0.5-K0.5.

### 2.2. Characterization of PVA/Marjoram/Kaolin Sponges

#### 2.2.1. FT-IR Analysis

**Figure 2:**
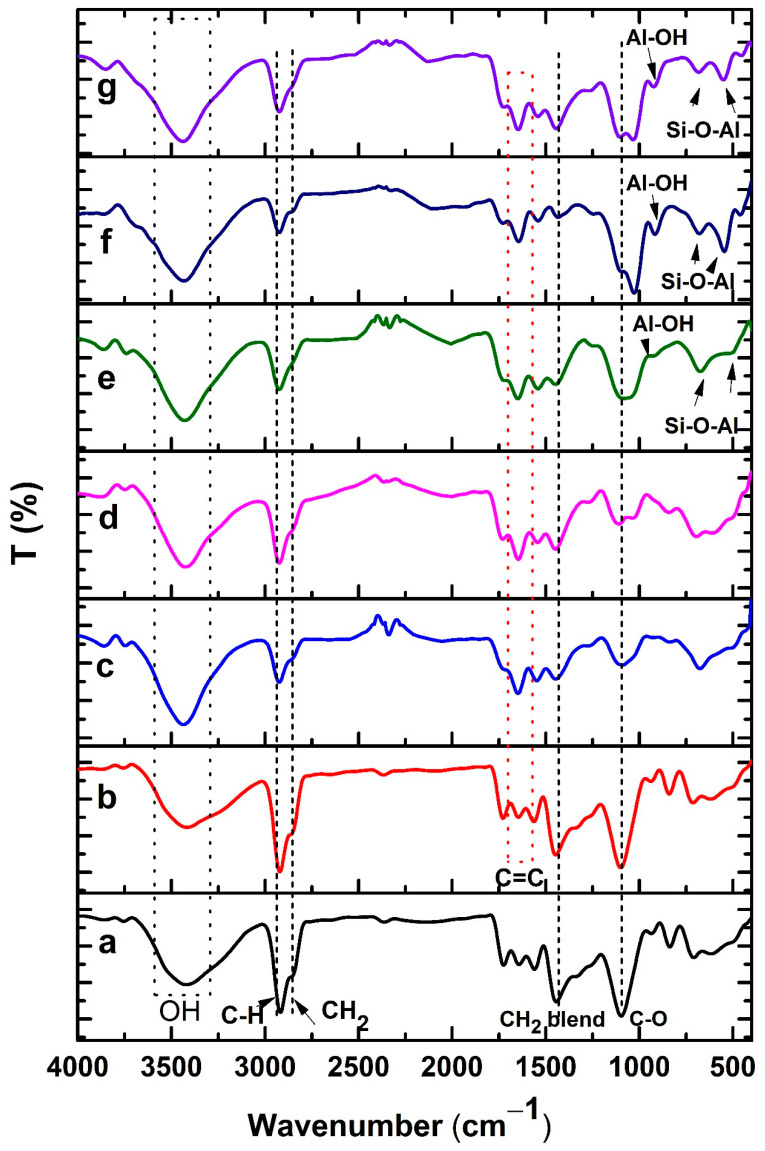
FT-IR spectra of (**a**) PVA, (**b**) PVA-M0.1, (**c**) PVA-M0.25, (**d**) PVA-M0.5, (**e**) PVA-M0.5-K0.1, (**f**) PVA-M0.5-K0.25, and (**g**) PVA-M0.5-K0.5 composite sponges.

As illustrated in Figure 2, the structures of the PVA sponge and the alterations of PVA composite sponges, including marjoram oil and kaolin with different contents, were studied by means of FT-IR. The appearance of characteristic bands at 3387 cm^−1^ is assigned to –OH on hydrogen bonds between –OH groups among PVA chains, bestowing the hydrophilic strengths to the PVA sponge [24]. The methyl groups’ asymmetrical and symmetrical C-H stretching vibration modes could be perceived in the PVA spectrum at 2926 cm^−1^. Moreover, the band at 2845 cm^−1^ corresponds to a methylene vibration band, whereas the distinct band at 1710 cm^−1^ is imputed to the stretching vibration band of the remaining acetyl carbonyl groups. The appearance of the band at 1450 cm^−1^ is attributed to asymmetrical and symmetrical CH bending vibrations of methyl groups [24].

Furthermore, a prominent band at 1118 cm^−1^ is the main indicator of the PVA structure [25], while a band at 1085 cm^−1^ corresponds to C–O–C. Incorporation of marjoram into PVA generated a new band at 1660 cm^−1^, which interacted with the stretching vibration band of the remaining acetyl carbonyl groups in the PVA. Evidently, this band turned out to be clearer at high concentrations of marjoram. On the other hand, the addition of kaolin to PVA/marjoram sponges led to the appearance of peaks at 920 to 940 cm^−1^ related to Al-OH vibration. Additionally, the bands at 530 and 789 cm^−1^ correspond to the Si-O-Al bond vibration band.

#### 2.2.2. SEM Analysis

**Figure 3:**
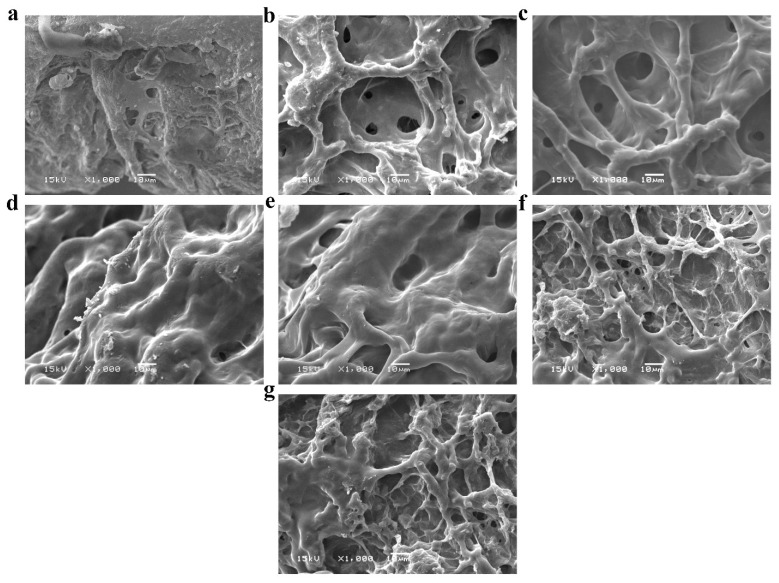
SEM images reveal surface morphologies of (**a**) PVA, (**b**) PVA-M0.1, (**c**) PVA-M0.25, (**d**) PVA-M0.5, (**e**) PVA-M0.5-K0.1, (**f**) PVA-M0.5-K0.25, and (**g**) PVA-M0.5-K0.5 composite sponges at a magnification of 1000×.

**Figure 4:**
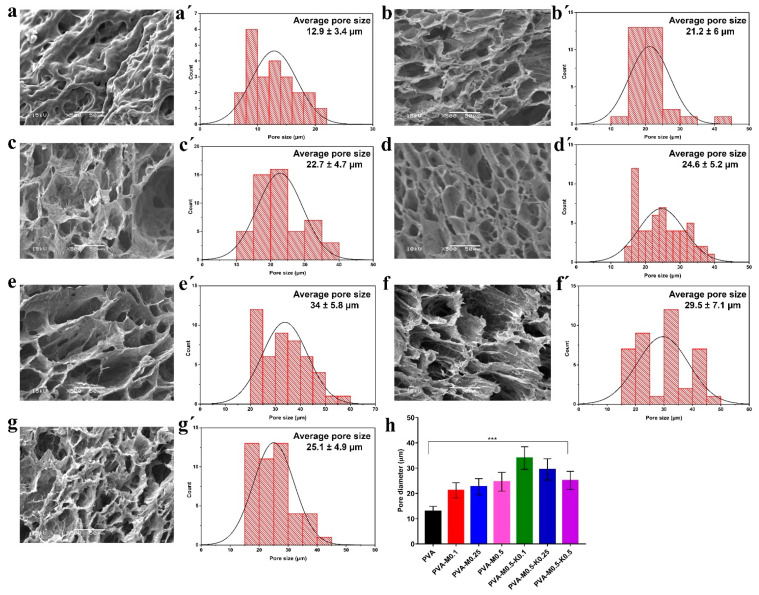
SEM images show cross-sectional and porous structures (**a**) PVA, (**b**) PVA-M0.1, (**c**) PVA-M0.25, (**d**) PVA-M0.5, (**e**) PVA-M0.5-K0.1, (**f**) PVA-M0.5-K0.25, and (**g**) PVA-M0.5-K0.5 composite sponges at a magnification of 500×. (**a’**–**g’**) illustrates the pore size distribution of PVA, PVA-M0.1, PVA-M0.25, PVA-M0.5, PVA-M0.5-K0.1, PVA-M0.5-K0.25, and PVA-M0.5-K0.5 composite sponges, respectively. (**h**) indicates the size of the average pores of PVA, PVA/marjoram, and PVA/marjoram/kaolin sponges. Results are presented as means ± SD (*** *p* < 0.001).

Microstructures of the synthesized sponges were probed by means of SEM. The morphological surface of the pure PVA sponges exhibited fewer pores than the PVA sponge composites. However, the PVA sponge composites amalgamated with various ratios of either marjoram (PVA/marjoram) or marjoram and kaolin (PVA/marjoram/kaolin) revealed three-dimensional structures interconnected with varied pore sizes in asymmetric arrangements as illustrated in Figure 3a–g. Furthermore, cross-sectional micrographs of the PVA/marjoram and PVA/marjoram/kaolin sponges exposed asymmetric neat structures with prominent three-dimensional interconnected networks as portrayed in Figure 4a–g. Furthermore, the sponge composites showed porous sponge layers with evident lamellar structures comparable to previously applied PVA sponge composites in wound healing with remarkable performances [13,26]. The sponge with a dense structure, on the other hand, is not favorable for hemostatic wound dressings, which necessitates high conductivity to the wound for absorbing wound surplus exudate and interacting with blood to enhance the hemostatic reaction [26,27].

Moreover, antibacterial and antioxidant wound dressings should firmly seal the wound to stall the bacterial invasion and mitigate the free radicals generated during the wound healing process [28]. Given these requisites, the development of highly porous sponges is indispensable to promote the attachment and proliferation of keratinocytes and fibroblasts during epithelialization, thereby stimulating angiogenesis at the wound bed [29]. Besides, such sponges prevent the dehydration of the wound, permitting the exchange of fluids and gases toward the wound and furnishing the corresponding cells with nutrients required for its propagation [27].

From the data in Figure 4a’–d’, it can be perceived that the extent of pore sizes of the developed sponges increased with the rise of marjoram ratios, recording 12.9 ± 3.4 µm, 21.2 ± 6 µm, 22.7 ± 4.7 µm, 24.6 ± 5.2 µm for PVA, PVA-M0.1, PVA-M0.25, and PVA-M0.5, respectively. These findings are most likely due to the lessening of molecular crystallizations of PVA, which act on the cross-linking sites of marjoram extract, leading to the diminution in crosslinking density in the case of PVA/marjoram sponges [30,31]. Furthermore, the amalgamation of various kaolin concentrations into PVA/marjoram augmented the pore sizes, reporting 34 ± 5.8 µm, 29.5 ± 7.1 µm, 25.1 ± 4.9 µm for PVA-M0.5-K0.1, PVA-M0.5-K0.25, and PVA-M0.5-K0.5, respectively, as presented in Figure 4e’,f’. As delineated in Figure 4h, the maximum pore size was determined for PVA-M0.5-K0.1, while the increase in kaolin ratios lowered the pore size. This might be attributed to the aggregation of kaolin with marjoram extract as a consequence of the emulsification of the marjoram oil.

These findings are consistent with those of earlier studies [31], which demonstrated the outstanding impacts of the wound dressings with pore sizes in a range of 19.5–36.7 μm. Moreover, previously fabricated Poly(ionic liquid)/PVA hydrogels based on PVA with pore sizes ranging from 10 to 30 μm exhibited an influential role during the wound healing of rats [13]. Furthermore, previous reports showed that the wound dressings with pore sizes ranging from 20 to 125 μm had a decisive function in furnishing the dermal cells with oxygen and necessary nourishments, thereby ameliorating the regeneration of skin tissues [32]. Moreover, it has been reported that the measurements of significant human cells extend over a range from 2 to 120 μm [12]. Collectively, the PVA/marjoram and PVA/marjoram/kaolin with pore sizes in the range of 21–34 μm could be encouraging sponge composites for further implementation as wound dressings.

#### 2.2.3. TGA

**Figure 5:**
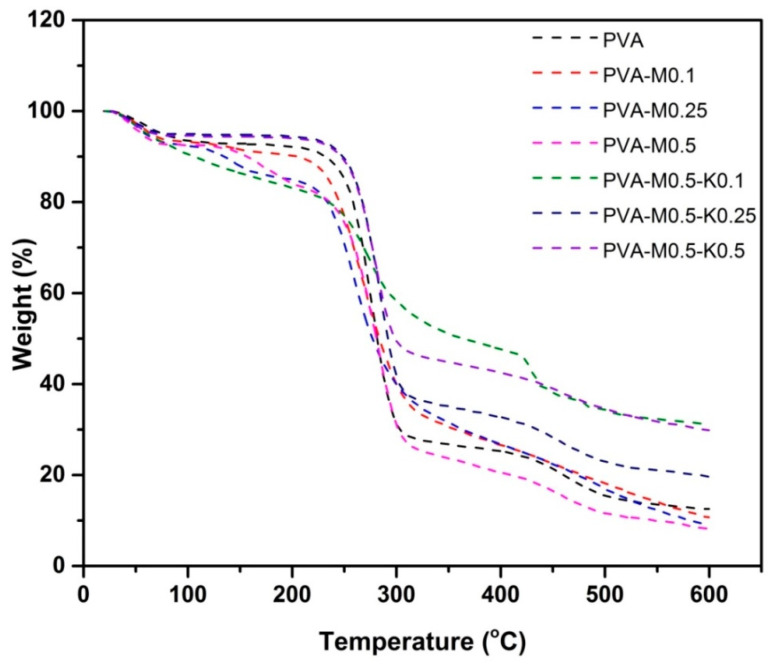
TGA charts of PVA, PVA/marjoram, and PVA/marjoram/kaolin composite sponges.

The thermal degradation behaviors of PVA and PVA/marjoram/kaolin sponges were investigated using TGA as shown in Figure 5. The figure charts represent several degradation steps of sponge composites. The initial stage started at ambient temperature, indicating the loss of piping water moisture content. Specifically, PVA neat sponges exhibited weight losses of 6.55% at 98.8 °C, PVA-M0.1 lost 6.76% at 73.84 °C, PVA-M0.25 lost 6.52% at 71.44 °C, PVA-M0.5 lost 6.16% at 67.67 °C, PVA-M0.5-K0.1 lost 5.98% at 67.5 °C, while PVA-M0.5-K0.25 and PVA-M0.5-K0.5 weight losses were 4.64% and 4.86%, respectively at 67.9 °C. Marjoram oil and kaolin had significant effects on the moisture contents of sponges, in which increase in marjoram or kaolin concentrations exhibited a significant decrease in the trapped solvent or water molecules. This might be attributed to the hydrophobic nature of marjoram oil, whereas in the case of kaolin, it could be explained by a decrease in the PVA content in the composite ratio, in which hydroxyl groups of PVA have a significant role in trapping moisture content. The combination of marjoram oil in the blend sponges resulted in new degradation characters perceived in peak between 109–200 °C with a magnitude weight loss of 3.89% for PVA-M0.1, 7.56% for PVA-M0.25 and 9.4% for PVA-M0.5. This could be attributed to the loss of volatile components of marjoram oil. It could also be recognized that this peak was absent in the sponge containing kaolin, which may be explained by the role of kaolin for stabilizing marjoram oil in the blend structure. Second weight loss for PVA sponges was observed from 226 °C to 314 °C with a weight loss of 62.5% corresponding to thermal decomposition of PVA backbone and eliminated water and carbon dioxide. The weight loss of this degradation stage was decreased by the addition of marjoram and more significantly by adding kaolin. Formulation of PVA blends with marjoram and kaolin stimulated the formations of the internal micro pore that can act as an interior atmosphere and trapped degraded byproduct carbon dioxide gases. The third degradation stage until 600 °C could be ascribed to the decomposition of organic residues. The remaining weight over 600 °C refers to the inorganic remains of kaolin, which did not degrade at this temperature.

#### 2.2.4. Gel Fraction, Swelling Profile, Hydrodegradation In Vitro, and Porosity Analyses

**Figure 6:**
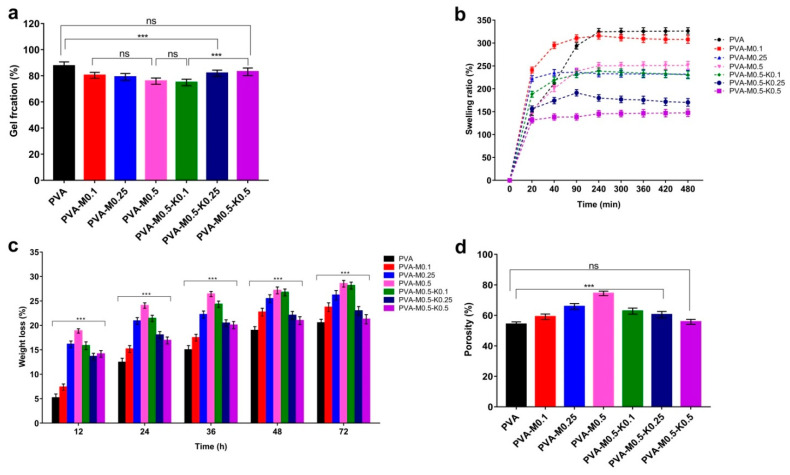
(**a**) Gel fractions, (**b**) Swelling characteristics, (**c**) In vitro weight loss, and (**d**) Porosity evaluations PVA/marjoram, and PVA/marjoram/kaolin composite sponges compared with the pure PVA sponge. Results are stated as means ± SD (*n* = 5) (*** *p* < 0.001, and (ns) points to non-significant difference).

Gel fraction properties of PVA/marjoram and PVA/marjoram/kaolin sponges were inspected to explore the influence of marjoram oil and marjoram alongside kaolin particles on gel formation, respectively. Figure 6a demonstrates that the introduction of marjoram oil into the PVA significantly dropped the gel fraction percentages, recording 87.5 ± 3.4%, 80.3 ± 2.4%, 79.2 ± 2.9%, and 76.8 ± 2.5% for PVA, PVA-M0.1, PVA-M0.25, and PVA-M0.5, respectively. However, the results showed no significant differences correlated with the increase of marjoram ratios. On the other hand, the gel fraction ratios were insignificantly lowered for the PVA-M0.5-K0.1 (74.9 ± 2.1%) compared to the entire PVA/marjoram groups. At the same time, the additional growth in kaolin concentrations significantly amplified the gel fraction ratios, reporting 82 ± 3.8% and 83 ± 2.9% for PVA-M0.5-K0.25, PVA-M0.5-K0.5, respectively. Therefore, it seems possible that these results are due to the manipulation influence of marjoram and kaolin particles on the structure of PVA. Additionally, these outcomes indicate that incorporating marjoram and kaolin into the PVA weakened the crosslinking density, which might further enhance the swelling behavior of PVA/marjoram/kaolin dressings [33]. It could thus reinforce the absorption aptitude of excess wound exudates by these respective sponges, hampering adverse complications.

The swelling capability of hemostatic dressings acts as a key factor for hemostasis and wound healing downstream by governing the bleeding, drugs’ release, degradation, and biological fluid absorption [34]. Accordingly, the 3-dimensional polymeric structures alongside the hydrophilic groups associated with the backbone of polymer chains improve the capacity of sponges to absorb great amounts of water without disintegration [35]. Therefore, the in vitro swelling characteristics of the developed sponges were analyzed, as displayed in Figure 6b. Swelling performances showed significant statistical differences (*p* < 0.001) in the swelling percentages for the entire sponges in relation to the PVA sponge. Furthermore, upon immersing the sponges in the water, quick swelling ratios were recorded for PVA/marjoram and PVA/marjoram/kaolin sponges with regard to the PVA. This is a very important property for hemostatic dressings in order to concentrate blood clotting determinants, thereby stimulating hemostasis [36]. Specifically, in the case of PVA/marjoram, the greatest water retention of 310 ± 9% was reported for PVA-M0.1 after 1.5 h among the other respective sponges. Furthermore, the increase in the ratio of marjoram oils resulted in significant reductions in the swelling manners, reporting 238 ± 11% and 236 ± 8% for PVA-M0.25, and PVA-M0.5 groups, respectively. This could be linked to the hydrophobic effect of oil ingredients [37].

On the other hand, the addition of kaolin particles at the lowest concretion for the PVA-M0.5-K0.1 sponge slightly influenced the swelling capacity, recording 233 ± 5%. Moreover, the statistical analyses exposed no significant variances in the PVA-M0.5-K0.1 sponge compared with the PVA-M0.25 and PVA-M0.5 groups. Nevertheless, the swelling ratios significantly decreased with the growth in kaolin levels. These findings could be attributed to the difference in the pore sizes, consistent with the data obtained from SEM analyses. Furthermore, the swelling ratios of the whole sponges levelled off after 4 h, accomplishing an equilibrium state. Additionally, the PVA-M0.1 group achieved the highest swelling ratio of 315 ± 12% after 4 h among the PVA/marjoram groups, while the greatest swelling ratio of 239 ± 7% was found for the PVA-M0.5-K0.1 sponge among the PVA/marjoram/kaolin sponges. Additionally, the graph shows that the similar results remained steady after 8 h. Previous studies reported the promotion of wound healing in vivo by applying wound dressings formulated on the basis of PVA and other biopolymers with different swelling percentages of 20%, 102% and 130% [13,38,39], highlighting the potential use of the PVA-M0.5-K0.1 sponge to ameliorate wound recovery.

The in vitro degradation of the PVA/marjoram and PVA/marjoram/kaolin sponges was studied by submerging them in PBS at 37 °C for predetermined times. As can be seen in Figure 6c, after 72 h of incubation, the PVA/marjoram sponges showed detectable weight losses of 23.7 ± 0.9%, 26.2 ± 1%, and 28.5 ± 0.6% for PVA-M0.1, PVA-M0.25, and PVA-M0.5, respectively. In contrast, the PVA group exhibited a weight loss of 20.5 ± 0.7%. These outcomes imply that the addition of marjoram oil promoted the degradation rate of sponges. On the other hand, the incorporation of kaolin alongside marjoram oil reduced the weight loss ratios, reporting 28.2 ± 0.8%, 23% ± 1, and 21.3 ± 0.8% for PVA-M0.5-K0.1, PVA-M0.5-K0.25, and PVA-M0.5-K0.5, respectively. This could be related to the aggregation of kaolin and marjoram, which could be perceived with the high concentration of kaolin. It is presumed that the in vitro degradation could impact the drug release represented by marjoram in this study, which is consistent with earlier studies [40,41]; thus, PVA-M0.5-K0.1 could be considered as the ideal wound dressing.

In aiming to appraise the water holding capacity of PVA/marjoram and PVA/marjoram/kaolin sponges, the porosity was tested. Figure 6d delineates the positive influence of marjoram oil on the porosity ratios for PVA/marjoram groups. The porosity for the PVA was 54.2 ± 2.4%, whereas PVA-M0.1, PVA-M0.25, and PVA-M0.5 reported porosities of 59.1 ± 2%, 65.8 ± 3.6%, and 74.4 ± 2.1%, respectively. This might be a result of the distortion impact of the marjoram oil on the internal structure of the polymer. Nonetheless, the introduction of kaolin particles into PVA-M0.5 lessened the porosity, and this decrease is correlated with the growth of the supplemented kaolin ratio. For PVA/marjoram/kaolin sponges, the porosity was determined to be 62.8 ± 3.9%, 60.5 ± 2.4%, and 55.8 ± 3.1% for PVA-M0.5-K0.1, PVA-M0.5-K0.25, and PVA-M0.5-K0.5, in respective order. These findings are in line with those of previous studies [42]. This performance might be related to the frequency of kaolin particles within the pores of sponges consuming some hydrogen bonds. Thus, the sponges turned out to be more compressed, and this obstructs the utilization of previously available pores [22]. The high porosity and water uptake competency of wound dressings are vital features for precluding microorganism invasion, expanding drug loading, and promoting dermal cell adherence and propagation. It could therefore ameliorate wound healing through preventing the prolongation of the inflammation phase.

In summary, swelling capacity, porosity, and biodegradation characteristics point to PVA-M0.5-K0.1 among the tested sponges for wound dressing applications.

### 2.3. Antibacterial Evaluation

**Figure 7:**
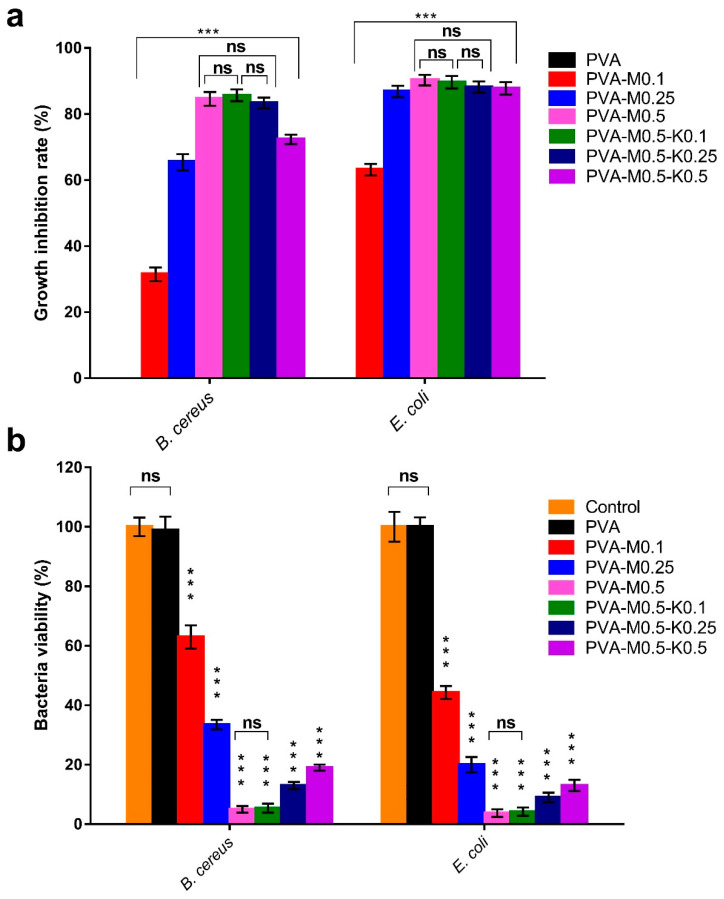
Antibacterial evaluations of PVA/marjoram and PVA/marjoram/kaolin composite sponges against *B. cereus* and *E. coli* compared to the pure PVA sponge adopting (**a**) growth turbidity method and (**b**) plate count method to determine the percentage of bacteria viability. Results are depicted as means ± SD (*n* = 5) (*** *p* < 0.001, and (ns) points to non-significant difference).

The antibacterial activity of wound dressing is paramount to preclude the growth of pathogenic microorganisms on the wound bed and even on the surface of the wound dressing itself, which retards the rejuvenation of skin tissues and might incite tissue maceration [39,43,44,45]. To this end, we bolstered the devised sponges with marjoram oil as one of the natural antibacterial products as an alternative to conventional antibiotics to prevent the emergence of multi-drug resistant bacteria [21]. The antibacterial performances of PVA/marjoram and PVA/marjoram/kaolin sponges against *B. cereus* and *E. coli* were estimated by the growth turbidity method, as shown in Figure 7a. A significant positive correlation between the antibacterial capacity of PVA/marjoram sponges and the increase in the marjoram ratios is discernible. Specifically, the pure PVA sponges revealed no activity with regard to the tested bacteria. Remarkably, the addition of marjoram bestowed the antibacterial potency on the PVA-M0.1, PVA-M0.25, and PVA-M0.5 sponges, recording 31%, 65%, and 85% with regard to *B. cereus*, respectively. Moreover, PVA-M0.1, PVA-M0.25, and PVA-M0.5 sponges exerted growth inhibitions of 63%, 87%, and 90% in relation to *E. coli*, respectively. The variations in antibacterial capacity could be ascribed to the dissimilarity of cell walls for Gram-positive and Gram-negative bacteria.

The introduction of kaolin into the sponges in terms of PVA/marjoram/kaolin sponges showed no substantial differences in antibacterial activity for the PVA-M0.5-K0.1 and PVA-M0.5-K0.25 groups compared with the PVA-M0.5 group. By contrast, the antibacterial activity of the PVA-M0.5-K0.5 group was significantly diminished. This phenomenon could stem from the aggregation of kaolin with marjoram, hampering the release of marjoram oil into the medium.

The antibacterial features of PVA/marjoram and PVA/marjoram/kaolin sponges were further investigated using a colony-forming unit. As illustrated in Figure 7b, comparable trends to the previous results could be perceived. Significantly, PVA-M0.5 sponges could inhibit 95% and 97% of *B. cereus* and *E. coli*, respectively. Furthermore, the antibacterial behaviors of the PVA-M0.5-K0.1 group revealed no statistical differences in comparison with the PVA-M0.5 group, reporting growth inhibition ratios of 95% and 97% toward *B. cereus* and *E. coli*, respectively. In contrast to these findings, the inhibition ratios of the examined bacteria significantly lessened for PVA-M0.5-K0.25 and PVA-M0.5-K0.5 sponges. The variance of these results compared to the previous approach for PVA-M0.5-K0.25 could be explained by the inevitable measurement of viable and dead bacterial cells by the spectrophotometer in terms of the growth turbidity method. In summary, these findings suggest that the PVA-M0.5-K0.1 sponge could be implemented to frustrate microbial infections and further ameliorate wound healing.

### 2.4. Total Phenolic Content and Antioxidant Evaluation

One of the detrimental bioburdens during wound healing is the overabundance of reactive oxygen species (ROS), provoking oxidative stress as a result of the phagocytosis mechanism [39,46]. This might incite lipid peroxidation, deactivation of vitals enzymes in addition to the damage of DNA, leading to impairment of wound healing and adjacent skin tissues [47]. Thus, the boost of wound dressing by antioxidant compounds is crucial to modulate the overabundance of ROS [29,48].

**Figure 8:**
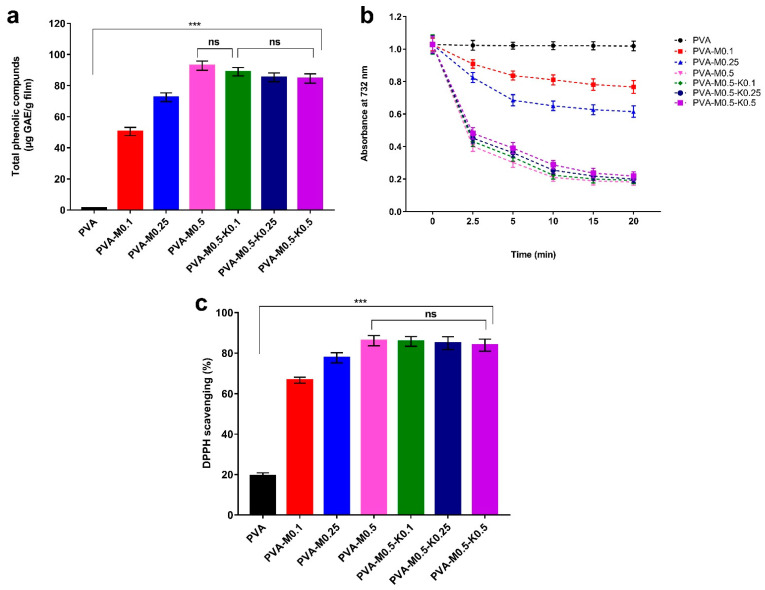
(**a**) Total phenolic compounds released from PVA/marjoram, and PVA/marjoram/kaolin composite sponges, (**b**) Time-dependent decolourization of ABTS^•+^ dye by PVA/marjoram, and PVA/marjoram/kaolin composite sponges, and (**c**) Scavenging competency of DPPH free radical by PVA/marjoram, and PVA/marjoram/kaolin composite sponges. Results are presented as means ± SD (*n* = 5) (*** *p* < 0.001, and (ns) points to a non-significant difference).

A striking attribute of essential oils is their containing various phenolic compounds, which exclusively endow them with vital biological activities, such as antioxidant characteristics, in order to scavenge reactive oxygen species (ROS). In this regard, it has been reported that marjoram oil contains phenolic acids and terpenoids [21]. To explore the efficiency of PVA/marjoram and PVA/marjoram/kaolin sponges to release the marjoram oil into the surrounding medium represented by the phenolic compounds, the total phenolic contents were determined subsequent to immersing the tested sponges in ethanol. Accordingly, the entire sponges exploited their structural integrity and released the corresponding phenolic mixtures. As can be seen from Figure 8a, phenolic compounds were not detected for the pure PVA sponge, which served as a negative control. For the composite sponges, there is a clear trend of increasing the phenolic contents with the rise in the marjoram oil for PVA-M0.1, PVA-M0.25, and PVA-M0.5 groups. The incorporation of kaolin into the sponges altered the release profile of marjoram oil; however, no significant reduction in the release profile was found for the PVA-M0.5-K0.1 sponge compared with the PVA-M0.5 sponge.

On the other hand, in comparison with the PVA-M0.5 sponge, the release profiles of phenolic contents were significantly diminished for PVA-M0.5-K0.25 and PVA-M0.5-K0.5 sponges. This manner could be ascribed to the adsorption of marjoram oil on the surface of kaolin particles. Concurrently, it is worth mentioning that there were no statistically significant differences in the release profile of phenolic contents for PVA-M0.5-K0.25 and PVA-M0.5-K0.5 groups in relation to the PVA-M0.5-K0.1 group.

Figure 8b displays the time-dependent decolorization of the ABTS^•+^ cationic radical by ethanol extracts of PVA, PVA/marjoram and PVA/marjoram/kaolin sponges. It could be discerned that the PVA sponge (control) exhibited slight ABTS^•+^ radical scavenging activity, which might be ascribed to the presence of hydroxyl groups along the PVA backbone. However, ABTS^•+^ radical scavenging activity was significantly enhanced by adding marjoram oil to PVA/marjoram groups. Besides, the amalgamation of kaolin in terms of PVA/marjoram/kaolin groups reduced the ABTS^•+^ radical scavenging capacity without a significant difference for PVA-M0.5-K0.1 compared to PVA-M0.5. Moreover, the statistical analyses demonstrated no significant differences in ABTS^•+^ radical scavenging capacity for PVA-M0.5-K0.25 and PVA-M0.5-K0.5 sponges in relation to the PVA-M0.5-K0.1 sponge. These results are in agreement with those obtained by measuring the total phenolic content in the previous section. Explicitly, the incidence of phenolic compounds in marjoram oil imparted an electron to ABTS^•+^, which further decolorized and converted to a neutral form [49,50,51].

To further evince the antioxidant potency of PVA/marjoram and PVA/marjoram/kaolin groups, an in vitro design system has been applied to estimate the studied materials’ capacity to eradicate free radicals based on DPPH assay. The mechanism of this assay depends on the scavenging of the stable free radical 1,1-diphenyl-2-picrylhydrazyl (DPPH) by reducing the DPPH violet color into yellow-colored diphenyl-picrylhydrazine as a consequence of accepting an electron from antioxidant compounds [52]. Figure 8c delineates the scavenging activity of the DPPH dye by PVA/marjoram and PVA/marjoram/kaolin sponges. The results are consistent with those presented in the ABTS^•+^ assay. Furthermore, the pure PVA sponges showed a mild scavenging ratio of the DPPH dye on account of hydroxyl groups. At the same time, there are positive associations between the scavenging ratios of DPPH and the increase in marjoram oil contents. Moreover, the introduction of kaolin exposed no significant difference of DPPH scavenging for the entire PVA/marjoram/kaolin groups with regard to the PVA-M0.5 sponge.

Together, these results evidently indicate the potential application of PVA/marjoram/kaolin sponges in wound healing, particularly the PVA-M0.5-K0.1 sponge, without any significant influence on the emancipation of phenolic compounds from marjoram oil.

### 2.5. Hemocompatibility, Thrombogenicity and Cytotoxicity Evaluations

**Figure 9:**
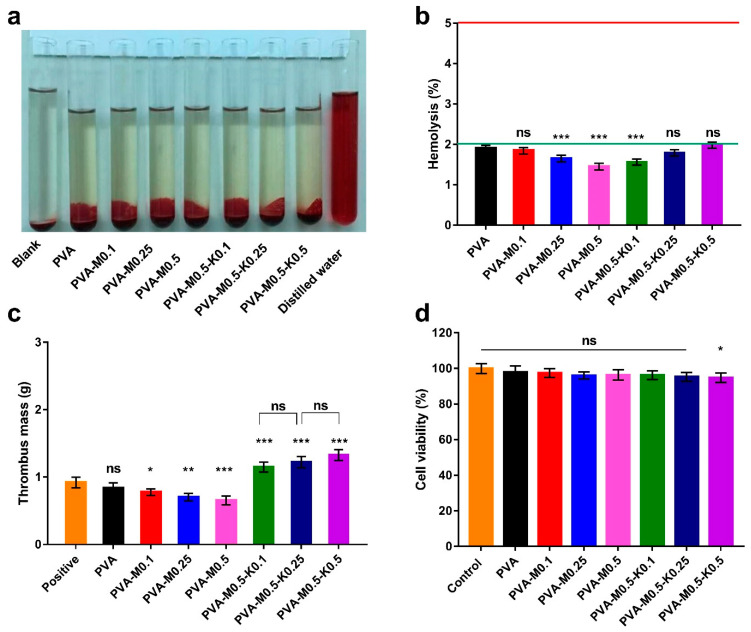
Photographs for the hemolysis test (**a**) and hemolytic percentages (**b**) reveal the hemocompatibility of PVA/marjoram and PVA/marjoram/kaolin composite sponges. (**c**) Thrombogenicity assessment for PVA/marjoram and PVA/marjoram/kaolin composite sponges demonstrate their capability to clot the blood through the formation of thrombus mass. (**d**) Cytotoxicity of PVA/marjoram and PVA/marjoram/kaolin composite sponges toward fibroblast cells show the safe behavior of the developed sponges. Results are presented as means ± SD (*n* = 6) (*** *p* < 0.001, ** *p* < 0.01, * *p* < 0.05, and (ns) points to a non-significant difference).

Hemocompatibility of biomaterials tailored to wound dressings, particularly hemostatic dressings, is an intrinsic property due to the unavoidable interactivity between blood and applied dressings [53,54]. The macroscopic photo in Figure 9a revealed the obvious variance in color between the seven sponge groups, the positive control (distilled water), and the negative control (PBS). Clearly, the entire sponge groups and the negative control emerged in yellow color without a significant difference. Conversely, the positive control tube appeared in red, implying the complete lysis of erythrocytes. The quantitative data of the hemolysis ratios for sponges PVA, PVA-M0.1, PVA-M0.25, PVA-M0.5, PVA-M0.5-K0.1, PVA-M0.5-K0.25 and PVA-M0.5-K0.5 were 1.9%, 1.84%, 1.65%, 1.45%, 1.56%, 1.79%, 1.98%, respectively, as provided in Figure 9b. It could be extrapolated that the tested sponges showed non-hemolytic activities (<2%) according to the American Society for Testing and Materials (ASTM F 756-00, 2000), implying the good hemocompatibility of the PVA/marjoram/kaolin sponges.

The thrombogenicity test was conducted to appraise the PVA/marjoram/kaolin sponge groups’ capacity to clot the blood, as illustrated in Figure 9c. Compared to the positive control, the thrombus formation was decreased for the PVA sponge; however, this diminution was not statistically significant. Therefore, this manner could be attributed to the hydrophilic trait of PVA. Nevertheless, it could be discovered that the introduction of marjoram oil substantially lessened thrombus formation. These results agree with those observed in prior studies [55,56], which elucidated this action by the presence of active phenolic compounds in marjoram oil, which hinder the aggregation of platelets. On the other hand, the supplementation of PVA/marjoram with different ratios of kaolin significantly escalated the weight of thrombus, which could be imputed to the influential blood clotting function of kaolin particles. Altogether, hemocompatibility and thrombogenicity findings suggest that PVA/marjoram/kaolin sponges could be significantly utilized as hemostatic wound dressings.

Cell compatibility test of wound dressings is crucial in order to assess the extent to which the fabricated sponges possess favorable biocompatibility in response to prevalent dermal cells, such as fibroblast, keratinocyte, and epithelial cells. During wound recovery, fibroblasts play a pivotal role in the construction of connective tissues, giving rise to the granulation of skin tissues and promoting skin remodeling [12,27,57]. Given this fact, alongside the cell interactions with wound dressings, we performed cytotoxicity investigations on fibroblast cells by the MTT assay. The cytotoxicity results exhibited no significant difference among the entire studied sponges, except for the sponge PVA-M0.5-K0.5 compared to untreated cells, as illustrated in Figure 9d. Thus, the feasible application of PVA-M0.5-K0.1 and PVA-M0.5-K0.25 sponges in wound dressings can be deduced from these findings since they could sustain the dermal cells in healthy behavior for achieving their functions properly.

Overall, these salient results advocate future in vivo wound healing studies for the PVA-M0.5-K0.1 sponge composite as an antibacterial and hemostatic wound dressing.

## 3. Materials and Methods

### 3.1. Materials and Bacterial Strains

PVA (Mw = 72 kDa) was purchased from ACROS Organics™, Carlsbad, CA, USA. Chinese marjoram oil and absolute ethanol were provided from Sinopharm Chemical Reagent Co., Ltd. (Beijing, China). Kaolin (hydrated aluminium silicate), MTT 3-(4,5-dimethylthiazol-2-yl)-2,5-diphenyltetrazolium bromide, sodium hydroxide, dimethyl sulfoxide (DMSO), and acid citrate dextrose solution (ACD) were obtained from Sigma–Aldrich (Chemie GmbH, Steinheim, Germany). Folin–Ciocalteu, gallic acid, 2,2-diphenyl-1-picrylhydrazyl (DPPH) and 2,2′azino-bis(3-ethylbenzothiazoline-6-sulphonic acid, ABTS) were purchased from Sigma-Aldrich Co., Ltd., St. Louis, MO, USA. Dulbecco’s Modified Eagle Medium (DMEM) and trypsin were purchased from Gibco (ThermoFisher Scientific, Waltham, MA, USA). Yeast extract, tryptone, and sodium chloride were received from Bioshop (Canada Inc., Ontario, CA, Canada).

*Bacillus cereus* (*B. cereus*) and *Escherichia coli* (*E. coli*), representing Gram-positive and Gram-negative bacteria, respectively, were used to study the antibacterial activities of the fabricated dressings. Prior to performing the antibacterial assay, the bacterial strains were refreshed via inoculating into LB medium containing g/L: NaCl 10 g, peptone 10 g, and yeast extract 5 g, and then incubated at 37 °C and 150 rpm for 18 h.

### 3.2. Methodology

#### 3.2.1. Preparation of Sponges

PVA/marjoram/kaolin sponge composites were fabricated adopting a freezing-thawing cycle approach in accordance with previously described procedures [58]. Briefly, mixtures of 5% (*w*/*v*) PVA, various concentrations of marjoram extract oil (0.1, 0.25, and 0.5 mL), and kaolin (0.1, 0.25, and 0.5 g) were thoroughly mixed prior to sonication and vortexing for 1 h. After that, the blends were poured into Petri dishes, followed by five successive cycles of freezing at −20 °C for 18 h and then thawing for 6 h at 25 °C. The sponges with different volumes of marjoram (0.1, 0.25, and 0.5 mL) were designated as PVA-M0.1, PVA-M0.25, and PVA-M0.5, respectively, in addition to a control sponge of PVA. Moreover, the sponge PVA-M0.5 was supplemented with various kaolin contents (0.1, 0.25, and 0.5 g) and labelled as PVA-M0.5-K0.1, PVA-M0.5-K0.25, and PVA-M0.5-K0.5, respectively. The developed sponges were frozen in liquid nitrogen for 10 min and then lyophilized for further examinations.

#### 3.2.2. Characterization of the Sponges

##### FT-IR Analysis

The alterations of the chemical structures of the designed sponges were investigated using a Fourier transform infrared spectrophotometer after thoroughly mixing a weight of 5 mg of each sponge with potassium bromide (KBr). The FT-IR equipment (Shimadzu 8400S, Kyoto, Japan) was programmed to probe each sponge 40 times at a range of 400–4000 cm^−1^ for 40 scans.

##### Morphological Examination and Thermal Analysis

To scrutinize the formulated sponges’ morphological variations, each sample was overlaid with a thin film of gold under vacuum conditions prior to surveying by scanning electron microscope (SEM, Joel Jsm 6360LA, Tokyo, Japan).

For thermal characterization, ∼5 mg of each film in a sealed aluminium pan was analyzed employing a thermal gravimetric analyzer (TGA, Shimadzu 50/50H, Kyoto, Japan) within a temperature range of 20–600 °C with a heating rate (10 °C/min) and a nitrogen flow of 30 mL/min.

##### Gel Fraction, Swelling Profile, In Vitro Degradation, and Porosity of Sponges

For gel fraction determination, the sponges were placed in a vacuum oven for 24 h at 50 °C until dried and then weighed. Thereafter, the sponges were re-swollen for 24 h in distilled water until an equilibrium swelling point for eliminating soluble PVA was obtained. The sponges were subsequently dried in a vacuum oven at 50 °C and weighed [59]. The experiments were conducted in five replicates, and the gel fractions were estimated using Equation (1) as follows:

Gel fraction (%) = (We/Wi) × 100(1)

where We and Wi refer to the weights of the dried sponge and swollen sponge, respectively.

The swelling capacities of sponges were appraised via the determination of their weights after immersing them into the water for time intervals. Approximately 1 g of a dried sponge was weighed and then dipped into 500 mL of distilled water. Dynamic Swelling was performed at 25 °C until reaching an equilibrium state. Each swollen sample was withdrawn at predetermined time points, and the water-adhered onto the surface was gently blotted using filter papers before weighing. Each experiment was replicated five times, and the swelling ratios were estimated using Equation (2) as follow:

Swelling ratio (%) = [(Ws − Wd)/Wd] × 100(2)

where (Ws) denotes the weight of the swollen sponge, while (Wd) refers to the weight of the sponge at the initial time.

To evaluate the porosity of the sponges, the measurements were performed following the procedure used by Yin et al. [60]. First, sponges were dried at 50 °C for 2 h in a vacuum oven and then the dried weights were estimated. Next, the samples were plunged for 4 h in absolute ethanol. Subsequently, the swollen sponges were blotted to eliminate the extra ethanol using filter paper and then weighed. Finally, the porosity analyses were conducted in five replicates and calculated according to the Equation (3):

Porosity (%) = [(W2 − W1)/pV] × 100(3)

where W1 and W2 refer to the weight of the sponge prior to and after being immersed in absolute ethanol, respectively, “V” represents the volume of the sponge, and “p” denotes the density of absolute ethanol.

To perceive the hydrolytic degradation of the devised sponges, dried sponges were weighed, immersed into 3 mL PBS (0.1 M, pH 7.4), and maintained at 37 °C. The sponges were then taken out at different time points and mildly wiped with soft papers to eliminate the excess water on the surface of sponges. Following this, the samples were dried under vacuum conditions and weighed. All experiments were conducted in five replicates.

#### 3.2.3. Antibacterial Analysis

The antibacterial evaluation of the prepared sponges was conducted adopting two approaches to determine optical densities and the colony-forming unit (CFU/mL). First, overnight bacterial cultures of *B. cereus* and *E. coli* were diluted in LB medium prior to adapting their turbidities were adapted following the McFarland 0.5 standard at 625 nm with 2 × 10^8^ CFU/mL [61,62]. Subsequently, 100 µL of the diluted bacterial cultures were inoculated into 10 mL LB medium, including 50 mg of tested sponges, before being incubated for 18 h at 37 °C and 150 rpm. In contrast, the bacterial cultures without sponges were set out as controls. After incubation, the antibacterial capacity was estimated by two approaches; first by estimating the inhibition of bacterial growth employing a spectrophotometer at 600 nm and calculating the ratio of bacterial growth inhibition were determined using Equation (4):

Bacterial growth inhibition (%) = [(ODc − ODi)/ODc)] × 100(4)

where ODc and ODi are the optical densities of bacterial cultures untreated and treated with a tested sponge, respectively.

For the second evaluation method, 50 µL of bacterial cultures were spread over LB agar plates and then maintained for 24 h at 37 °C before ascertaining the colony-forming unit (CFU/mL). Thus, both antibacterial tests were accomplished in five replicates.

#### 3.2.4. Total Phenolic Content

The phenolic contents of the formulated films were assessed based on reducing the Folin-Ciocalteu reagent from yellow to blue colored compound. First, 50 mg of each membrane was submerged into 5 mL of ethanol to extract the marjoram oil content in the membrane [63]. Afterwards, 0.5 mL of sponge supernatant was put into 2.0 mL of Folin–Ciocalteu reagent (10%, *v*/*v*), followed by adding 2 mL sodium carbonate solution (7.5%, *w*/*v*). The blend was maintained at 50 °C for 5 min, and the absorption was then gauged at 760 nm by means of a spectrophotometer. The determinations were replicated five times and estimated in relation to the stranded curve for gallic acid solutions (0–100 µg).

#### 3.2.5. Antioxidant Activity Determination

##### ABTS^•+^ Radical Scavenging Assay

For the ABTS radical scavenging assessment, the radical cations were prompted by the reaction of an aqueous solution of K_2_S_2_O_8_ (3.30 mg) in water (5 mL) with ABTS (17.2 mg). Then, the resultant bluish-green radical cation solution was kept overnight below 0 °C under dark conditions. Later, 1 mL of the solution was diluted to a final volume of 60 mL with distilled water and marked as the ABTS^•+^ solution. The samples were extracted as presented above in the estimation of total phenolic content. Following this, 0.1 mL of each sponge leachate was put into 2.0 mL of ABTS^•+^ solution. The ABTS^•+^ evaluation was implemented five times, and the absorption was appraised at 730 nm at various time points.

##### DPPH Radical Scavenging Activity

The antioxidant properties of the sponge leachates were assessed by adapting the 2,2-diphenyl-1-picrylhydrazyl (DPPH) approach [43,64]. Accordingly, 6 mg of DPPH were dissolved in 50 mL methanol (0.3 mM), and then a volume of 2.5 mL of each sponge extract and 2.5 mL of the DPPH solution was thoroughly mixed. Next, the tube was incubated at room temperature for 20 min under dark conditions. Afterwards, the decolorization of the dye was quantified at 517 nm using a spectrophotometer. The reactions were replicated five times, and inhibition percentages of radicals were computed by the following Equation (5):

DPPH scavenging (%) = [(Ac − As)/Ac)] × 100(5)

where Ac is the absorbance of the control DPPH solution and As is the absorbance of the sponge extract after reaction with the DPPH solution.

#### 3.2.6. Hemocompatibility of the Sponges

In order to investigate the hemocompatibility of the formulated sponges, the hemolysis tests were executed as previously demonstrated with minor adaptations [65]. Anti-coagulated blood was prepared for this determination by adding 1 mL of anticoagulant acid citrate dextrose solution (ACD) to 9 mL of blood. Prior to commencing the direct contact between blood and the tested membranes, about 1 cm^2^ of each film was plunged in phosphate buffer solution (PBS, pH 7.0) for 72 h at 37 °C. Following that, the PBS was poured out before immersing the sponges in 1 mL of ACD blood and keeping the tubes at 37 °C for 3 h. For preparing negative and positive control tubes, the equivalent volumes of the ACD blood were added to 7 mL of PBS and water, respectively. The tubes were carefully inverted three times every 30 min to preserve the appropriate contact of the films with the blood. Subsequently, each liquid was carefully moved to new tubes and clarified via centrifugation for 15 min at 200 rpm. The hemoglobin released by hemolysis was estimated at 540 nm employing a spectrophotometer (Model Ultrospec 2000). All determinations were implemented in five replicates, and the hemolysis ratio was computed using Equation (6):

Hemolysis (%) = [(ODm − ODn)/(ODp − ODn)] × 100(6)

where ODm is the absorbance value of a tested sponge, ODn is the absorbance value of the negative control, and ODp is the absorbance value of the positive control.

#### 3.2.7. Thrombogenicity Test

A gravimetric method was applied, as described earlier, to ascertain the amounts of formed thrombus over the surface of the fabricated sponges [57]. ACD blood was prepared as demonstrated above. Membranes were plunged into PBS for 48 h at 37 °C. On completion of incubation time, the PBS was then poured out, and the ACD blood was positioned over the examined materials. At the same time, positive control was set out by applying the equivalent amount of ACD blood to an empty Petri dish.

For prompting the clotting reaction of blood, 20 µL of a 10 M calcium chloride solution was put onto the sponges. After 45 min, the reactions were terminated by adding 5 mL H_2_O. Subsequently, the clots were firmly attached with an additional 5 mL of a 36% formaldehyde solution and dried with tissue papers before weighing. Thrombogenicity examinations were repeated five times.

#### 3.2.8. Cytotoxicity Test of the Sponges

The cellular toxicity of the membranes on NIH 3T3 (mouse fibroblast cells) was appraised adopting MTT [3-(4,5-Dimethythiazol-2-yl)-2,5-Diphenyltetrazolium Bromide] method as demonstrated earlier with some adaptations [66,67].

The NIH 3T3 cells were cultivated in Dulbecco’s modified Eagle’s medium (DMEM), dissolved in 10% fetal bovine serum and fostered at 5% CO_2_ and 37 °C with a humidity of 85% in a CO_2_ incubator. Then, 0.5% trypsin was applied to detach the fibroblasts, and the cells were thereafter seeded at 5 × 10^3^ cells/well in a 96-well plate. The plate was then incubated in the CO_2_ incubator as mentioned above for 24 h.

Concurrently, 30 mg of each sponge was sterilized by immersing in 70% ethanol, subjected to UV for 45 min, and transferred into a 24-well plate containing 1 mL of DMEM at 37 °C for 24 h. Subsequently, the medium was discarded from the plate containing fibroblast cells and replaced with 100 µL of the membrane’s leach, while the control cells were supplied with 100 µL of standard DMEM medium. After that, the 96-well plate was incubated for 24 h before washing the cells with PBS three times. Then, the MTT test was commenced by adding 20 µL of MTT solution (5 mg/mL in serum-free medium) to each well, and the plate was then maintained in the CO_2_ incubator for 3 h at 37 °C. Subsequently, the MTT solution was replaced with 200 µL of dimethylsulfoxide (DMSO) for each well. Finally, the plate was shaken at 100 rpm for 5 min, and the absorbance values were estimated at 570 nm by means of a microtiter plate reader. The investigation was carried out in six replicates for each sponge, and the viable ratio of fibroblast cells was evaluated following Equation (7):

Cell viability (%) = (Am/Ac) × 100(7)

where (Am) refers to the absorbance of cells doped with tested membrane, while (Ac) points to the absorbance of untreated cells.

#### 3.2.9. Statistical Analysis

GraphPad Prism software (V. 5) was employed to analyze the statistical significance of the entire data. One-way and two-way analyses of variance (ANOVA) with Tukey’s multiple comparison tests were thus applied. The entire determination values are expressed as means ± SD, and they were significantly considered at *p*-value < 0.05, where *n* = 5, except for the cytotoxicity studies (*n* = 6).

## 4. Conclusions

In summary, novel sponges based on PVA boosted by marjoram essential oil and kaolin were successfully designed to frustrate massive bleeding and bacterial infection, which could further accelerate full-thickness wound healing. PVA/marjoram/kaolin sponges exhibited noticeable porous and lamellar structures. The amalgamation of marjoram and kaolin into PVA augmented the pore size of the devised sponges, thus encouraging cell attachment and proliferation. Moreover, they demonstrated great water absorption, which supports their competency to govern the hemorrhage quickly. PVA/marjoram/kaolin sponges presented an outstanding performance in scavenging free radicals as antioxidant sponges and revealed high antibacterial activity in relation to pathogenic bacteria. Furthermore, manifested thrombogenicity, hemocompatibility, and cellular compatibility were corroborated for the developed sponges. Thus, the results clearly indicate the PVA-M0.5-K0.1 sponge for future consideration as a hemostatic and antibacterial wound dressing. Therefore, future in vivo studies are justified to determine the extent of PVA-M0.5-K0.1 to enhance cutaneous wound restoration in cases of bleeding and microbial infections.

## Data Availability

The datasets generated during the current study are available from the corresponding authors upon request.
